#  Capital estrangeiro e mudança estrutural no mercado privado de serviços de saúde brasileiro 

**DOI:** 10.1590/0102-311XPT171222

**Published:** 2023-09-25

**Authors:** Felipe Duvaresch Kamia, Marco Antonio Vargas

**Affiliations:** 1 Universidade Federal Fluminense, Niterói, Brasil.; 2 Fundação Oswaldo Cruz, Rio de Janeiro, Brasil.

**Keywords:** Sistemas de Saúde, Instituições de Saúde, Complexo Econômico-Industrial da Saúde, Economia da Saúde, Financiamento de Capital, Health Systems, Health Facilities, Health Economic-Industrial Complex, Health Economics, Capital Financing, Sistemas de Salud, Instituciones de Salud, Complejo Económico-Industrial de la Salud, Economía de la Salud, Financiación del Capital

## Abstract

Este artigo analisa o processo de transformação estrutural no mercado privado de serviços de saúde brasileiro a partir dos anos 2000, com ênfase na crescente participação de fundos financeiros e do capital estrangeiro no processo de expansão e consolidação do setor. A análise do movimento de ingresso do capital estrangeiro nos serviços e planos de saúde no Brasil foi desenvolvida a partir da construção de uma base dados com um total de 297 operações patrimoniais envolvendo empresas com atividades em serviços de saúde, inclusive operadoras de planos e seguros de saúde e administradoras de benefícios em saúde. A análise dessas operações evidencia que o afluxo de capital estrangeiro foi fundamental para viabilizar a centralização de capital em determinadas empresas e catalisar o processo de concentração e transformação estrutural do setor de serviços de saúde ao longo das últimas duas décadas. Conclui-se que o acirramento da disputa intercapitalista no mercado de serviços de saúde levou à emergência de grandes corporações no mercado e a novos modelos de negócio, com destaque especial para o surgimento de redes verticalizadas de atendimento (operação de planos, serviços hospitalares, ambulatoriais, de diagnóstico e tratamento e de atenção básica).

## Abstract (en)

This article analyzes the process of structural transformation within the Brazilian private health services market since the 2000s, with emphasis on the growing participation of financial funds and foreign capital in the process of expansion and consolidation of the sector. The analysis of the movement of foreign capital into health services and plans in Brazil was developed from the construction of a database with a total of 297 equity operations involving companies with activities in health services, including companies operating health plans and insurance and companies administering health benefits. The analysis of these operations shows that the influx of foreign capital was fundamental to enable the centralization of capital in certain companies and catalyze the process of concentration and structural transformation of the health services sector over the last two decades. We concluded that the intensification of the intercapitalist dispute within the health services market led to the emergence of large corporations and new business models, with special emphasis on the emergence of verticalized care networks (operation of plans, hospital services, outpatient services, diagnosis and treatment, and primary care).

## Resumen

Este artículo analiza el proceso de transformación estructural en el mercado privado de servicios de salud brasileño, a partir de los años 2000, con énfasis en la creciente participación de fondos financieros y del capital extranjero en el proceso de expansión y consolidación del sector. El análisis del movimiento de ingreso del capital extranjero en los servicios y planes de salud en Brasil fue desarrollado a partir de la construcción de una base datos con un total de 297 operaciones de capital de empresas con actividades en servicios de salud; inclusive las empresas operadoras de planes y seguros de salud y las empresas administradoras de beneficios en salud. El análisis de esas operaciones muestra que la entrada de capital extranjero fue fundamental para viabilizar la centralización de capital en determinadas empresas y catalizar el proceso de concentración y transformación estructural del sector de servicios de salud a lo largo de las últimas dos décadas. La conclusión que el recrudecimiento de la disputa intercapitalista dentro del mercado de servicios de salud llevó a la emergencia de grandes corporaciones en el mercado y nuevos modelos de negocio, con destaque especial para surgimiento de redes verticalizadas de atención (operación de planes, servicios hospitalarios, ambulatorios, de diagnóstico y tratamiento y de atención básica).

## Introdução

Este artigo discute o processo de transformação estrutural no mercado privado de serviços de saúde brasileiro, com o objetivo de produzir novas evidências sobre a formação de grupos econômicos na saúde. Para isso, foi construída e analisada uma base de dados com operações patrimoniais envolvendo empresas com atividades em serviços de saúde, incluindo as operadoras de planos de saúde, entre 1999 e 2018. A análise permitiu evidenciar que o capital estrangeiro e empresas financeiras foram importantes para capitalizar firmas que protagonizaram o processo de conglomeração no mercado privado de serviços de saúde brasileiro.

A análise da dinâmica econômica na área da saúde constitui um grande desafio, tanto do ponto de vista acadêmico como no tocante aos seus aspectos político-normativos, tendo em vista a articulação entre saúde e desenvolvimento. Com base na perspectiva do Complexo Econômico-Industrial da Saúde (CEIS) ^1^, que alia a economia política com a saúde coletiva, o mercado é entendido como um espaço econômico de disputa entre capitais em permanente mutação, derivado da influência dos diversos interesses e forças políticas, públicas e privadas, que o compõem.

As atividades do CEIS, a base econômica e material da saúde, representam cerca de 9% dos empregos diretos formais; 1/3 do esforço de pesquisa do país e 9% do produto interno bruto (PIB) brasileiro ^1^. Grande parte desse fluxo econômico passa por atividades privadas de saúde. O mercado privado de serviços de saúde, em 2019, foi responsável por cerca de 4,6% do PIB e 3,5% dos empregos formais no Brasil ^2^.

Apesar de a abordagem do CEIS destacar a importância das diversas formas de organização do sistema de saúde sobre a dinâmica dos demais subsistemas, poucos estudos focaram especificamente a dinâmica econômica do subsistema de serviços de saúde ^3^. O artigo contribui para a literatura da dinâmica econômica dos sistemas produtivos e de inovação em saúde, em especial o CEIS, pelo enfoque no movimento das grandes empresas privadas que atuam no mercado de saúde brasileiro e por aproximar a literatura do CEIS com trabalhos do campo da saúde coletiva sobre as relações público-privadas do sistema de saúde do país.

A mudança estrutural no mercado de serviços de saúde, a partir da perspectiva do CEIS, relaciona-se a transformações em três dimensões inter-relacionadas: (i) as dimensões econômica, produtiva e tecnológica, com a introdução de inovações que geram oportunidades, por meio de novas formas de organização do financiamento, da produção (e realização) dos serviços de saúde e da apropriação das rendas geradas nesses serviços; (ii) as dimensões demográfica, epidemiológica e socioeconômica da população, que se traduzem em demandas de cuidado em saúde e na capacidade de pagamento das famílias; (iii) a dimensão institucional, que envolve desde as características dos modelos assistenciais adotados, as políticas públicas para a área da saúde e a ação das agências regulatórias, até aspectos culturais, como a percepção do direito à saúde pela sociedade; e (iv) a interação com os demais subsistemas dos CEIS ^1^^,^^4^.

As transformações nessas dimensões devem ser apreendidas no contexto do processo histórico, marcado por rupturas e continuidades, de evolução do capitalismo, do desenvolvimento brasileiro e, mais especificamente, da formação do sistema de saúde brasileiro, que influenciam, em um movimento dialético e não determinístico, as possibilidades de acumulação privada na saúde e, consequentemente, sua articulação com o Sistema Único de Saúde (SUS). Acelerado a partir da virada do século, esse conjunto de transformações propiciou a emergência de novas estratégias empresariais e, como consequência, um vigoroso processo de reconfiguração do mercado. A conglomeração e a concentração do setor são expressões mais visíveis desse processo. Nessa perspectiva, o estudo da dinâmica do mercado privado de assistência à saúde no Brasil passa a ser importante para entender a evolução da relação público-privada em saúde e suas consequências para o acesso universal à saúde no país ^5^.

### O mercado privado de saúde no Brasil

Do ponto de vista institucional e organizacional, o sistema de saúde brasileiro se revela como uma rede complexa de prestadores e compradores de serviços públicos e privados, financiada majoritariamente por estes últimos recursos, que competem entre si ^6^. Por um lado, as empresas privadas de saúde oferecem serviços de atenção à saúde mediante o pagamento direto ou dos planos e seguros privados, além de ofertarem serviços para o SUS, remunerados pelo setor público. Por outro lado, parte da produção dos serviços ofertada por operadoras privadas de planos de saúde é realizada por estabelecimentos públicos ou serviços privados conveniados ao sistema público ^6^. Essa combinação público-privada específica vai muito além das relações de compra e venda de serviços de saúde e envolve um conjunto de relações que abrangem desde o sistema de formação e qualificação de profissionais de saúde até o desenvolvimento e a difusão de inovações nesse sistema ^7^^,^^8^^,^^9^. A constitucionalização do direito à saúde como dever do Estado e direito do cidadão, inclusive, atua como um resseguro para o sistema privado, na medida em que funciona como um garantidor em última instância do sistema de saúde, reduzindo os riscos de atuação das empresas privadas no setor ^5^.

O mercado de serviços privados de saúde compreende as formas de produção, gestão e realização de atividades de atenção à saúde humana, com o objetivo de atender a determinada demanda ou clientela restrita. Ele é composto por um grupo heterogêneo de agentes, que variam em termos de porte, funções assistenciais e nível de complexidade tecnológica, natureza jurídica (pública, privada com ou sem fins lucrativos), além da possibilidade de aceitar diversos modelos de pagamento (por meio do SUS, de planos de saúde ou gasto direto). O ponto primordial que caracteriza o mercado privado de saúde é a restrição do acesso mediante o pagamento pela utilização dos serviços.

Pode-se diferenciar as atividades do mercado em dois grupos. O primeiro se refere aos estabelecimentos, com ou sem fins lucrativos, que prestam serviços de atenção à saúde diretamente à população ou a outros estabelecimentos de saúde. O grupo denominado “serviços de saúde” pode ser subdividido entre hospitais, ambulatórios e serviços de apoio ao diagnóstico e tratamento (SADT). O segundo grupo abrange planos de saúde, seguros de saúde e administradoras de benefícios em saúde, denominados operadoras de planos de saúde (OPS). Apesar de serem compostas por diversos tipos de organizações empresariais, como as cooperativas médicas, medicina de grupo, planos de autogestão e outras formas de associação, essas atividades são reguladas desde 1999 pela Agência Nacional de Saúde Suplementar (ANS).

Os principais desafios econômicos para as OPS são aumentar a adesão dos planos de saúde, reduzir a taxa de sinistralidade, cumprir as exigências regulatórias e conter o crescimento dos custos de acesso aos serviços privados de saúde. No segmento dos serviços, os principais desafios são a introdução de inovações no modelo de atendimento (hospitais-dia, telemedicina, *home care*, oferta de serviços ambulatoriais customizados ou de nicho, como o Grupo Oncoclínicas), o aumento da eficiência operacional e a ampliação da remuneração junto às OPS e ao SUS ^10^. Em uma perspectiva generalizante, o grande desafio das empresas no mercado privado de serviços de saúde é a ampliação dos mercados e contenção dos custos na prestação dos serviços de saúde, por meio da introdução de inovações como as supracitadas.

A relação entre a transformação das empresas privadas de serviços de saúde e as transformações político-institucionais do sistema de saúde brasileiro, especialmente no setor de hospitais e OPS, foi objeto de estudo em diversos trabalhos no campo da saúde coletiva ^5^^,^^7^^,^^9^^,^^11^^,^^12^^,^^13^. O SUS é a expressão da ideia de saúde como uma parte integrante de um amplo sistema de “*seguridade social pública, universal, garantidora de direitos de cidadania e que não tergiversava acerca da responsabilidade do Estado na sua implementação*” ^14^ (p. 446). Um problema de pesquisa importante, portanto, tem sido investigar o crescimento das empresas privadas de saúde ao longo das últimas décadas, a despeito da inclusão da saúde como direito do cidadão e dever do Estado, na *Constituição Federal* de 1988, e da criação do SUS.

As políticas públicas de saúde dos anos 1960 e 1970 foram determinantes para moldar a relação público-privada no sistema de saúde brasileiro. As reformas impostas durante a ditadura militar, de caráter previdenciário, individual e assistencialista, transferiram a função provedora de serviços de saúde para a iniciativa privada e estabeleceram a política de contratação de serviços de saúde de terceiros pelo Estado ^5^^,^^7^^,^^9^. Por meio da orientação das políticas públicas que criavam demanda para o setor privado, e de subsídios diretos e indiretos para estimular a criação de oferta pelo setor privado, as empresas privadas de serviços de saúde experimentaram intenso crescimento ^11^^,^^12^^,^^13^. Concomitantemente, os interesses ligados a esses setores ganharam força e passaram a influenciar de forma crescente as políticas públicas na área da saúde, preservando e incentivando a livre iniciativa privada no mercado na saúde, mesmo após a criação do SUS ^9^.

A trajetória das empresas privadas que atuam no mercado de serviços de saúde, sob a perspectiva das relações público-privadas no sistema de saúde brasileiro, pode ser dividida em três ciclos ^15^. O primeiro corresponde ao sistema de saúde previdenciário vigente no Brasil entre os anos 1960 e 1980. Nesse período, surgem as empresas pioneiras, comandadas por médicos (com ou sem fins lucrativos), financiadas mediante o reinvestimento dos próprios recursos ^15^ e das políticas públicas de crédito subsidiado ^12^^,^^13^.

O segundo ciclo (década de 1990) é o período de consolidação dos grandes grupos médicos empresariais nacionais. Observa-se um rápido crescimento de planos empresariais e individuais no contexto de implementação do SUS, incentivado pelos subsídios ao atendimento da demanda via saúde suplementar ^9^^,^^12^, sob baixa regulamentação. O ciclo é marcado por uma crescente associação com o capital financeiro, pelo surgimento de grupos hospitalares, pelo intenso processo de terceirização e, em alguns submercados, como o dos laboratórios de análises clínicas, pela concentração e internacionalização ^11^.

A institucionalização e a regulamentação do mercado de saúde suplementar no país no fim dos anos 1990, que, entre outras medidas, também autoriza a participação do capital estrangeiro nas OPS, marcam o início do terceiro ciclo. Como diversas operadoras tinham redes de atendimento próprias, na prática, a lei abriu de forma disfarçada a possibilidade da participação do capital estrangeiro em serviços de saúde ^16^. Em 2015, a *Lei nº 13.097*^17^ alterou novamente os limites ao capital estrangeiro no mercado de serviços privados de saúde brasileiro. De sua proibição, salvo em casos previstos na lei (OPS), passa-se a uma abertura ampla e irrestrita desse mercado ao capital estrangeiro ^18^.

Tais transformações nos planos produtivo e institucional devem ser analisadas em conjunto com o processo de evolução do regime de acumulação capitalista. A transformação do regime de acumulação capitalista tem sido denominada frequentemente como financeirização ^19^^,^^20^^,^^21^^,^^22^, entendida como o aumento dos motivos financeiros, mercados financeiros, agentes financeiros e empresas financeiras na operação das economias nacionais e internacionais ^21^, ou como um novo padrão sistêmico da riqueza, marcado pela importância crescente de ativos financeiros na definição, gestão e realização da riqueza e a introjeção da lógica financeira nos processos decisórios dos agentes econômicos ^20^.

Os sistemas de saúde, e mais especificamente o mercado privado de assistência à saúde, têm sido fortemente influenciados pela difusão do processo de financeirização, tanto em termos globais ^22^^,^^23^ como nacionais ^24^^,^^25^, muitas vezes com impacto negativo para o acesso e a equidade ^23^. Esse movimento foi incentivado pela agenda dos organismos multilaterais, que, desde os anos 1990, têm promovido a agenda de crescimento dos interesses privados em espaços anteriormente vinculados ao setor público-estatal ^23^^,^^26^ e mecanismos de financiamento por meio do mercado de capitais ^27^.

O processo de financeirização tem influenciado a atuação de grupos econômicos e provocado alterações em diversas atividades do CEIS brasileiro ^25^^,^^28^^,^^29^^,^^30^^,^^31^^,^^32^. As empresas de saúde financeirizadas “*detêm um poder de arbitragem crescente, capaz de criar mercados e nichos de mercado para produtos e serviços e influenciar na valorização e desvalorização de ativos de natureza diversa incluindo, no caso da assistência, insumos para a saúde*” ^24^ (p. 414).

Os estudos sobre a financeirização em países periféricos e no mercado da saúde brasileiro, entretanto, apresentam diversos desafios teóricos. As empresas financeirizadas nacionais, incluindo as que operam no mercado de assistência à saúde, possuem características qualitativas e quantitativas distintas em comparação aos pares internacionais, sugerindo a necessidade de aprofundar estudos sobre o processo de concentração das empresas do setor, seu padrão de financiamento e de aplicação dos recursos obtidos no mercado financeiro ^31^^,^^33^^,^^34^.

## Metodologia

O movimento de ingresso do capital estrangeiro nos serviços e planos de saúde no Brasil foi analisado a partir da construção de uma base de operações patrimoniais envolvendo empresas com atividades em serviços de saúde, inclusive as OPS ^3^. A base contempla um total de 297 operações, que ocorreram entre os anos de 1999 e 2018, sendo que cada uma delas pode envolver a aquisição de uma ou mais empresas alvo.

Nas operações patrimoniais, parte do capital social de uma companhia é transferida para outra. Destacam-se as operações de fusão, incorporação, aquisição majoritária, aquisição minoritária e as ofertas públicas de ação iniciais (IPO - *initial public offering*) ou secundárias. Uma fusão é a união de duas empresas, de porte semelhante, que resulta na dissolução destas e na consequente criação de uma nova empresa. A incorporação é a aquisição de uma empresa alvo por uma empresa adquirente, que tem como resultado a dissolução da empresa alvo e sua incorporação dentro do capital da adquirente. Uma aquisição majoritária é a aquisição de parte do capital de uma empresa alvo que resulte no controle de mais 50% de seu capital social ao final da operação. Quando a aquisição resulta no controle inferior a 50% do capital da empresa alvo, é definida como minoritária. Finalmente, uma IPO é a venda de parte do capital social de uma empresa em uma bolsa de valores.

Os dados das operações patrimoniais utilizadas para construção da base foram obtidos junto à plataforma Zephyr (https://www.bvdinfo.com/en-us/our-products/data/greenfield-investment-and-ma/zephyr), organizada pela Orbis BvD, em outubro de 2019. Foram extraídas as operações completas ou assumidas como completas no período 1999 a 2018, envolvendo empresas com atividades no Brasil no segmento dos serviços de saúde e planos, seguros e intermediadoras de benefício em saúde.

A Orbis BvD classifica o setor de atividade das empresas através dos códigos NAICS 2017 (*North American Industry Classification System* - Sistema de Classificação da Indústria Norte-Americana). Cada empresa tem um código de atividade principal, referente ao seu *core business*, e um ou mais códigos de atividade secundários relevantes para ela. Por exemplo: uma empresa pode identificar “planos de saúde, operadoras e intermediadoras de seguros de saúde” como sua atividade principal, mas ter como atividades secundárias “hospitais” e “empreendimentos imobiliários”. Foram selecionadas empresas com atividades hospitalares, ambulatoriais, SADT, OPS e outros serviços de atenção à saúde, considerando os códigos de atividades dos capítulos 62 (*Health care and social assistance* - Saúde e assistência social) e outros selecionados dos capítulos 52 (*Finance and insurance* - Finanças e seguros) e 54 (*Professional, scientific, and technical services* - Serviços profissionais, científicos e técnicos) do NAICS 2017.

As operações patrimoniais foram classificadas de acordo com a origem do capital da empresa compradora. A categoria “nacional” representa o conjunto de operações cuja adquirente é residente no país, e cujo controlador não é identificado como estrangeiro. A aquisição do Hospital Nove de Julho pela Amil, em 2008, é um exemplo de operação nacional.

A operação foi classificada como “capital estrangeiro” quando a adquirente era uma firma não residente no Brasil, ou era subsidiária de empresa estrangeira com registro no Brasil. A aquisição do grupo NotreDame Intermédica pela Bain Capital, em 2014, e a aquisição do Hospital e Maternidade Madre Theodora pela Amil, em 2014, quando a Amil era controlada pela UnitedHealth, são exemplos de operações classificadas como “capital estrangeiro”. A origem do capital nas operações que não exibiam informações sobre as empresas adquirentes foi classificada como “indefinido”. Parte do capital identificado como estrangeiro nas transações patrimoniais pode ser fruto de capitais de origem nacional transnacionalizados (p.ex.: fundos sediados em paraísos fiscais). Entretanto, considera-se que, a partir do momento que o capital nacional foi transnacionalizado, ele assume características distintas do capital de origem nacional, radicado no Brasil.

Uma operação patrimonial pode ser realizada por uma, duas ou mais firmas. Uma rede de hospitais abre seu capital na bolsa de valores. Uma empresa de seguros de saúde adquire uma operadora de planos de saúde, com atividades em hospitais, ambulatórios, serviços de diagnóstico e tratamento. Três fundos de *private equity* adquirem ações de uma empresa de serviços de diagnóstico. Dois hospitais realizam uma fusão. Em cada um desses casos, a participação de ao menos uma empresa brasileira com atividades em serviços ou seguros de saúde foi considerada como o critério de seleção para a base.

Algumas operações não exibiam informações sobre as empresas adquirentes envolvidas. Para cada IPO, foi possível identificar e corrigir a participação de investidores estrangeiros na operação. Os valores associados aos demais subscritores do IPO, incluindo fundos institucionais, foram classificados como “indefinido”, dada a impossibilidade de identificar a origem do capital desses agentes nos documentos acessados. Muitas operações de captação realizadas por empresas médias ou pequenas também não continham informações do investidor.

## Resultados

Os dados sobre fluxo de investimento direto no país (IDP), divulgados pelo Banco Central do Brasil ^35^, que mede o ingresso do IDP por não residentes, mostram que, entre 2001 e 2014, o afluxo de investimento direto estrangeiro em empresas de serviços de saúde totalizou USD 138 milhões, o que indicaria uma pequena entrada de recursos. Após a abertura ao capital estrangeiro, observou-se um forte afluxo de investimento direto no país na área da saúde, que alcançou USD 1.337 milhões. Nos anos subsequentes, o movimento perde intensidade, mas se mantém em patamares mais elevados do que os observados antes da abertura.

**Figura 1:**
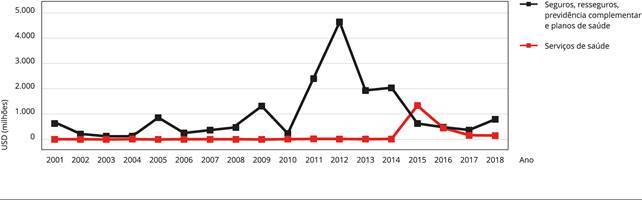
Investimento estrangeiro direto no mercado de serviços de assistência à saúde brasileiro.

Quando consideramos também os segmentos de seguros, resseguro, previdência complementar e planos de saúde, os dados do Banco Central apresentam entradas de capital estrangeiro com valores expressivos, especialmente na primeira metade da década de 2010. Os elevados valores nos grupos supramencionados ^18^^,^^31^ sugerem que a entrada do capital estrangeiro foi expressiva mesmo antes de 2015. O alto valor registrado em 2012 nesses setores, por exemplo, parece refletir o ingresso de capital estrangeiro proveniente da venda do grupo Amil pela UnitedHealth. De forma semelhante, os valores observados em 2011, 2013 e 2014 podem estar associados ao ingresso de capital estrangeiro em OPS (Figura 1).

Fonte: elaboração própria, com dados do Banco Central do Brasil ^35^.

A forma de apresentação dos dados do Banco Central, no entanto, torna opaca a atuação do capital estrangeiro no processo de mudança estrutural observado nos mercados de saúde privada brasileiros até 2015. A agregação dos dados com o setor de seguros, resseguros e previdência complementar, em conjunto com a impossibilidade de identificar as operações individualmente, dificulta uma associação direta.

Os resultados apresentam novas evidências, baseadas em microdados ao nível da empresa, que reforçam a hipótese de que o capital estrangeiro já exercia papel relevante no mercado de serviços de saúde desde a primeira metade da década de 2000. Adicionalmente, os dados apontam que o capital de origem estrangeira exerceu papel importante no financiamento do processo de mudança estrutural no mercado de serviços de saúde.

Entre 1999 e 2018, foram registradas 297 operações patrimoniais envolvendo empresas com atividades nos setores de serviços de saúde ou em seguros de saúde no Brasil. O tipo de operação mais comum foi a aquisição majoritária (194), seguido das operações de aquisição minoritárias (64). Foram identificadas oito IPO e 25 ofertas secundárias para aumento de capital. Observou-se uma fusão e a formação de quatro *joint ventures* no segmento de planos de saúde dentários.

**Figura 2:**
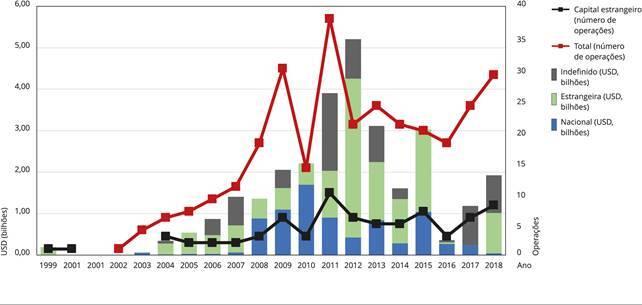
Evolução anual das operações patrimoniais e do volume financeiro envolvendo empresas de serviços e seguros de saúde no Brasil, de acordo com a origem do comprador (1999-2018).

O valor do negócio foi declarado em 165 operações (55%) dentre as selecionadas. Entre 1999 e 2018, essas operações movimentaram USD 29,3 bilhões, em valores corrigidos para 2018. O montante foi decomposto em três categorias, de acordo com a origem do capital da empresa adquirente. Quase metade do valor negociado (47%, USD 14 bilhões) está associada ao “capital estrangeiro”. Cerca de 27% do valor (USD 7,9 bilhões) foi identificado como “capital nacional” e 25% do valor negociado (USD 7,4 bilhões) tinha origem “indefinida”. A Figura 2 apresenta a evolução do volume financeiro anual negociado nas operações patrimoniais identificadas de acordo com a origem do capital (eixo vertical à esquerda) e a frequência de operações identificadas (eixo vertical à direita do gráfico).

Fonte: elaboração própria, com dados da Orbis ^36^ e do sistema de consulta *online* da Comissão de Valores Mobiliários (CVM) ^37^. Nota: valores reais para preços de 2018 (Índice de Preços ao Consumidor - IPC).

Nota-se uma aceleração da frequência e do volume financeiro movimentado nas operações patrimoniais no segmento de serviços e seguros de saúde a partir dos anos 2000. Esse movimento atinge seu auge em 2012, quando as operações movimentaram USD 5,2 bilhões pela venda da Amil para o grupo UnitedHealth pelo valor de USD 3,5 bilhões (origem do capital estrangeira) e pelas duas operações de venda de ações da Qualicorp, que movimentaram, no conjunto, USD 950 milhões (origem do capital indefinida), com o objetivo de remunerar o investimento do grupo Carlyle na empresa.

A Tabela 1 apresenta uma tabulação dos valores envolvidos nas operações patrimoniais. As operações nas quais a empresa compradora foi classificada como “capital estrangeiro” foram menos frequentes, mas apresentaram valores médios muito superiores às operações realizadas com capital nacional. Enquanto as 74 aquisições majoritárias realizadas por empresas de capital nacional mobilizaram USD 7,1 bilhões (média de USD 96 milhões por operação), as 14 aquisições majoritárias com capital estrangeiro movimentaram, igualmente, USD 7,1 bilhões (média de USD 500 milhões por operação).

**Tabela 1:** Informações das operações patrimoniais com valor declarado por origem do capital e tipo de operação.

	Frequência (valor declarado)	Valor total	Média	DP
Conjunto das operações				
Aquisição	89	14.262.991,20	160.258,30	426.194,90
Aquisição minoritária	44	8.219.727,00	186.812,00	217.493,20
IPO	8	3.531.943,00	441.492,90	257.148,90
*Joint venture*	1	1.116,90	1.116,90	-
Recompra de ações	1	7.195,10	7.195,10	-
Aumento de capital	22	3.349.007,00	152.227,60	271.414,60
Total	165	29.371.980,10	178.012,00	355.191,00
Operações com capital nacional				
Aquisição	74	7.138.703,30	96.469,00	186.987,20
Aquisição minoritária	15	788.434,60	52.562,30	59.319,80
*Joint venture*	1	1.116,90	1.116,90	-
Recompra de ações	1	7.195,10	7.195,10	-
Aumento de capital	1	1.724,90	1.724,90	-
Total	92	7.937.174,80	86.273,60	170.553,20
Operações com capital estrangeiro *				
Aquisição	14	7.116.547,40	508.324,80	936.919,60
Aquisição minoritária	16	4.053.514,40	253.344,70	208.957,00
IPO	8	2.758.904,00	344.863,00	221.660,00
Aumento de capital	2	56.332,60	28.166,30	4.386,50
Total	40	13.985.298,40	349.632,50	579.971,40
Operações com origem do capital indefinida *				
Aquisição	1	7.740,50	7.740,50	
Aquisição minoritária	13	3.377.777,90	259.829,10	276.790,20
IPO	7	773.039,00	110.434,10	45.635,00
Aumento de capital	19	3.290.949,50	173.207,90	287.244,40
Total	40	7.449.506,90	186.237,70	256.341,70

DP: desvio padrão; IPO: ofertas públicas de ação iniciais (*initial public offering*).

Fonte: elaboração própria, com dados da Orbis ^36^ e do sistema de consulta *online* da Comissão de Valores Mobiliários (CVM) ^37^.

Nota: valores em milhares de dólares, atualizados para o valor real em 2018.

* Devido às normas do mercado financeiro brasileiro, foi possível identificar, para cada IPO, a participação de investidores estrangeiros na operação.

Quando desconsideramos a aquisição da Amil pela UnitedHealth, em 2012, por USD 3,5 bilhões (49% do valor total das operações com capital estrangeiro), a média dessas transações é reduzida para USD 277 milhões. O valor se mantém superior à média das aquisições com capital nacional, mas próximo ao valor médio das aquisições minoritárias com capital estrangeiro, que foi de USD 253 milhões.

A Tabela 2 evidencia o intenso processo de consolidação no mercado de serviços de saúde no Brasil por meio das operações patrimoniais realizadas no setor e permite destacar características importantes desse processo. Ela apresenta uma matriz com o número de operações realizadas de acordo com a atividade principal da empresa compradora (eixo vertical) e o conjunto de atividades da empresa que teve ações adquiridas (eixo horizontal).

**Tabela 2:** Tabulação das operações patrimoniais de acordo com a atividade principal da empresa adquirente e atividade principal das empresas alvo (1999-2018).

Comprador	Alvo				
	Hospital	Ambulatórios	SADT	Suplementar	Outras atividades em saúde *
Hospital	23	0	1	1	0
Ambulatórios	1	9	0	0	3
SADT	10	2	48	0	8
OPS	5	13	0	17	7
Outros	1	4	4	0	6
Setor financeiro (exceto OPS)	13	19	25	42	42

OPS: operadoras de planos de saúde e/ou administradoras de benefícios; SADT: serviços de apoio ao diagnóstico e tratamento.

Fonte: elaboração própria, com dados da Orbis ^36^ e do sistema de consulta *online* da Comissão de Valores Mobiliários (CVM) ^37^.

Nota: em 7 operações foram adquiridas mais de uma empresa com códigos de atividade principal distintos, o que leva a soma de operações a totalizar 304 observações (p.ex.: a Odontoprev adquiriu, em 2011, a Odontoserv, Prontodente e Sepao. Enquanto a primeira é classificada como laboratório de serviços odontológicos, as outras duas foram classificadas como OPS).

* As “outras atividades em saúde” são compostas por seguradoras com atividades de seguros de saúde (38 operações); laboratórios de testagem (9), serviços de pesquisa & desenvolvimento (3), empresas de programação, coleta e processamento de dados (3), bancos comerciais (2), distribuidores de produtos de saúde (2), centro de assistência a idosos (1), serviços de consultoria (1), *holdings* (1) e serviços de construção e manutenção (1).

A maior parte das operações entre as empresas do mercado de serviços de saúde pode ser caracterizada como horizontal, na medida em que foram destinadas a obter alvos com a mesma atividade da empresa adquirente (diagonal principal da Tabela 2). No entanto, enquanto os hospitais focaram em operações horizontais, as empresas de SADT e OPS realizaram operações verticais dentro do subsistema de serviços do CEIS ou diversificaram suas atividades.

Observa-se um grande volume de operações de empresas do setor financeiro não vinculadas à saúde em operações patrimoniais destinadas a alvos com atividades hospitalares (13), ambulatoriais (19), de SADT (25), empresas com atividades no segmento de planos de saúde (42) ou empresas com outras atividades principais, mas que também atuavam em serviços de saúde (42). A maior parte dessas operações foi classificada como “operações minoritárias” (39%), nas quais não houve transferência do controle.

As operações minoritárias comandadas pelo capital financeiro somaram USD 7,9 bilhões (27% do valor total movimentado no período). Os resultados reforçam a hipótese de que esse tipo de transação, comandado por empresas financeiras, subsidiou o processo de conglomeração do setor, propiciando os recursos necessários para as empresas do mercado privado de saúde realizarem operações patrimoniais.

O Quadro 1 apresenta o detalhamento das operações patrimoniais do Grupo Dasa, de forma a ilustrar o movimento concreto das empresas de saúde. Após a injeção de capital por parte do fundo de *private equity* Patrimônio, em 1999, a Dasa inicia um conjunto de operações patrimoniais, adquirindo outros laboratórios de diagnóstico. Em 2004, a empresa abre o capital (IPO) e a Patrimônio encerra suas operações para rentabilizar a posição. A injeção de capital adicional propiciada pelo IPO e ofertas secundárias de ações (*follow-on*) propiciaram uma segunda rodada de aquisições. Em 2010, entretanto, a Amil realiza uma operação de troca de ações com a Dasa, abrindo espaço para o ingresso da família controladora da Amil na Dasa. Após a venda da Amil para a UnitedHealth, a família adquire o controle do Grupo Dasa e fecha o capital da empresa. Em 2021, segundo o ranking do *Valor Econômico*, o Grupo Dasa foi a segunda maior empresa de serviços de saúde do Brasil e a 99º maior empresa brasileira.

**Quadro 1:** Descrição das operações patrimoniais do Grupo Dasa (1999-2018).

ANO	CAPTAÇÃO/OPERAÇÃO ESTRATÉGICA	AQUISIÇÃO
1999	Patrimônio (*private equity*) adquire 49% da Delboni Auriemo, que passa a ser Diagnósticos da América S.A.	Laboratório Lavoisier
2000		Bronstein
2001		Lamina
2003		Laboratório Curitiba Santa Casa
2004	Abertura de capital (IPO); *private equity* encerram posições para rentabilizar operação	Elkis e Furlanetto; CRL; Presmed
2005		Laboratório Pasteur; Frischmann Aisengart; Alvaro
2006	Oferta pública de ações (*follow-on*)	LABPasteur; MedLabor; Vita; Atalaia
2007		CientíficaLab; Med Imagem
2008		Cedimax; Brafer; Clira; Digirad; Cedic; Cedilab; Centro Médico de Imagenologia; e Ressonância Magnética de Cuiabá
2009		Unidade Cearense de Imagem
2010	Troca de ações e acordo operacional entre Dasa e Amil *	Laboratório Exame; Image Memorial
2011		Laboratório Sergio Franco; Previlab; Cytola; Cerpe
2014	Reestruturação do Grupo Dasa Oferta pública de aquisição de ações: antigos controladores da Amil assumem controle do Grupo Dasa	
2015	Oferta pública de aquisição de ações (OPA); fechamento de capital	
2016		Laboratórios Gaspar, Leme e Gilson Cidrim
2017		Salomão Zoppi
2018		Laboratórios Oswaldo Cruz, Biomed, Sawana & Giana

IPO: ofertas públicas de ação iniciais (*initial public offering*).

Fonte: elaboração própria, com dados da Orbis ^36^ e do sistema de consulta *online* da Comissão de Valores Mobiliários (CVM) ^37^.

* A troca de ações foi feita entre o Grupo Dasa e o Grupo MD1, empresa detentora do controle do grupo Amil na ocasião.

## Discussão

O artigo agrega novas evidências sobre o crescimento dos grupos econômicos no mercado da saúde via operações patrimoniais. Os resultados reforçam a hipótese de que as operações patrimoniais no mercado de capitais permitiram a capitalização de grupos econômicos da saúde junto a empresas financeiras para promover o crescimento por meio de outras operações patrimoniais no mercado doméstico. Adicionalmente, suportam a hipótese de que a entrada do capital estrangeiro no setor de serviços de saúde brasileiro já vinha ocorrendo anos antes da aprovação da lei que libera sua atuação no setor de forma quase irrestrita ^15^^,^^16^.

Ao considerar o conjunto de operações patrimoniais, incluindo aquisições minoritárias lideradas por empresas financeiras, foi possível evidenciar um processo contínuo de entrada do capital financeiro nas empresas privadas de serviços de saúde. Tomando o valor das operações como uma *proxy* do porte da empresa adquirida, os resultados sugerem que o capital estrangeiro busca empresas de maior porte, enquanto as operações nacionais estariam mais vinculadas a um processo de conglomeração do mercado interno, adquirindo empresas de menor porte.

O aporte de capital por parte de empresas financeiras em empresas de capital fechado propicia caixa para remunerar antigos sócios e financiar sua expansão mediante operações patrimoniais. O ciclo de crescimento remunera os novos investidores, normalmente por meio da abertura do capital da empresa (IPO) e de ofertas secundárias (*follow-on*). Muitas vezes, entretanto, essas operações também envolvem um aumento de seu capital, financiando um novo ciclo de expansão através de operações patrimoniais.

A adoção dessa estratégia pode levar à redução de indicadores de alavancagem da dívida, utilizados com métricas da financeirização, mas não significa que as empresas estejam menos financeirizadas. Se o aporte de capital gerar aumento na quantidade de ações, por exemplo, e a empresa realizar operações patrimoniais com o recurso, o processo de crescimento via atração de fundos financeiros pode gerar redução na relação entre dívida e patrimônio líquido.

O capital levantado junto às empresas financeiras subsidiou um movimento de aquisições horizontais (resultando em maior concentração no setor de atividade) e verticais, levando à formação de redes de atendimento integradas e de diversificação (por meio da aquisição de empresas não relacionadas ao mercado de serviços de saúde).

Os resultados também sugerem que a participação do capital nacional se deu de forma mais associada do que subordinada aos fluxos de capital estrangeiro no mercado de serviços de saúde. Este teve um papel fundamental para capitalizar as empresas de saúde e financiar o processo de conglomeração do setor. A transferência do controle dos grandes grupos para o capital estrangeiro (sinalizada pela posse, direta ou indireta, de mais de 50% da estrutura acionária), no entanto, foi uma exceção, e não a “regra” do processo.

O controle da maior parte das empresas que lideraram o movimento de conglomeração se manteve com investidores nacionais, em muitos casos com acionistas das famílias dos mesmos grupos que participaram do processo de empresariamento da saúde nas décadas anteriores, corroborando resultados de outros trabalhos ^32^. A atualização da base de dados sobre operações patrimoniais para captar o intenso movimento de fusões e aquisições que se iniciou a partir de 2018-2019 deve verificar se o padrão se mantém associativo ou se tornou-se mais subordinado.

O comportamento das maiores empresas privadas do setor de saúde brasileiro difere substancialmente das estratégias das maiores empresas privadas de saúde globais (majoritariamente dos Estados Unidos), nas quais o controle acionário é pulverizado. O exemplo da trajetória do Grupo Dasa, após movimento de injeção de capital de ex-controladores da Amil, evidencia que o movimento concreto das empresas depende de questões específicas do processo de formação do mercado da saúde brasileiro, assim como da própria forma de atuação dos grupos econômicos nacionais e da saúde.

Os resultados evidenciam que a dominância financeira não implica, necessariamente, substituição de investimentos produtivos por financeiros ^22^. Em um ambiente marcado por fortes oportunidades de crescimento via conglomeração, a financeirização das empresas de serviços de saúde esteve mais associada à captação de recursos do que a estratégias especulativas no mercado de capitais.

## Considerações finais

Os desafios provocados pelas transformações econômicas, sociais e institucionais levaram à emergência de novos modelos de negócio no mercado privado de assistência à saúde brasileiro. O processo de financeirização ou dominância financeira nas empresas não financeiras esteve associado ao aumento no uso de operações patrimoniais ^22^. Ao se converterem em instrumentos frequentes e rotineiros no processo de crescimento das empresas, podem carregar informações relevantes sobre a evolução dessas firmas e do mercado.

Apesar das luzes lançadas sobre o processo de conglomeração e formação de grandes grupos econômicos na saúde, o esforço realizado ilumina apenas parcialmente esse processo. Ao considerar apenas as operações patrimoniais como evidência do processo de mudança estrutural, podemos deixar de perceber movimentos importantes realizados por empresas que não adotaram essa estratégia empresarial. A contribuição deste estudo deve se somar a outras pesquisas, que têm analisado o problema a partir de perspectivas distintas. Ao longo das últimas décadas, por exemplo, um grupo de instituições filantrópicas tem apresentado ritmo de crescimento intenso, se constituindo como parte das maiores empresas de saúde do Brasil. Investigar os fatores ligados ao desenvolvimento dos grandes grupos, portanto, segue como uma agenda de pesquisa relevante.

A análise histórica permite observar que o crescimento do mercado privado de saúde não é um processo natural. Historicamente, no Brasil, ele demandou intenso apoio do setor público, mediante a garantia de compras públicas, além do subsídio, da demanda e da oferta privada. Além de utilizar recursos públicos que poderiam ser mobilizados para investimentos no SUS, o crescimento do mercado privado de saúde sedimenta uma lógica mercantil no acesso à saúde, afastando da população a garantia do acesso universal, integral e equânime à saúde.

Além da introdução de inovações e da manutenção dos subsídios tributários, uma estratégia liderada pelo setor privado para expandir o mercado privado de assistência à saúde tem sido ampliar a segmentação do acesso. O *lobby* para autorizar a oferta de planos com cobertura limitada para a população sem capacidade de pagamento para os planos atuais ou a obrigação da oferta de planos empresariais para todos os trabalhadores formais são exemplos dessas agendas.

Em um cenário de estagnação econômica, as possibilidades de crescimento do setor privado demandam uma intervenção ainda maior do setor público. Aprofundar a compreensão dos fatores de crescimento do setor privado e da relação público-privado em saúde, portanto, é fundamental para compreender os novos desafios e oportunidades que surgem para viabilização do SUS de forma integral, equânime e universal.
